# Communication Difficulties and Associations With Health Literacy, Medication Beliefs, and Adherence in Multiple Sclerosis

**DOI:** 10.1002/brb3.71749

**Published:** 2026-09-07

**Authors:** Sawsan Khdair, Walid Al‐Qerem, Dunia Basem, Judith Eberhardt, Lama Sawaftah

**Affiliations:** ^1^ Department of Pharmacy, Faculty of Pharmacy Al‐Zaytoonah University of Jordan Amman Jordan; ^2^ Department of Psychology, School of Social Sciences, Humanities and Law Teesside University Middlesbrough UK

## Abstract

**Introduction:**

Communication difficulties are a common but under‐recognized feature of multiple sclerosis (MS) and can affect quality of life, social participation, and engagement with health care. The extent to which communication functioning is associated with health literacy, medication beliefs, and adherence in MS remains poorly understood.

**Methods:**

This cross‐sectional study included 307 adults with MS recruited from Al‐Bashir Hospital in Amman, Jordan. Participants completed validated Arabic versions of the Communication and Language Assessment Questionnaire for Multiple Sclerosis (CLAMS), the Health Literacy Survey‐12 (HLS‐Q12), the Beliefs about Medicines Questionnaire (BMQ), and the Medication Adherence Report Scale (MARS‐5). Sociodemographic data were collected. Quantile regression analysis was used to examine associations between communication functioning and health literacy, medication beliefs, adherence, and sociodemographic factors.

**Results:**

Communication difficulties were commonly reported, particularly problems with word finding, maintaining a train of thought, remembering information, and following conversations. Lower health literacy scores were significantly associated with higher CLAMS scores, indicating greater communication difficulties. Higher medication concern scores were also independently associated with poorer communication functioning. Lower educational attainment was linked to higher CLAMS scores. Medication adherence, necessity beliefs, age, sex, employment status, and income were not significantly associated with communication functioning in the adjusted model.

**Conclusion:**

Communication difficulties are prevalent among adults with MS and are closely linked to health literacy and medication‐related concerns. Addressing these factors may support more effective communication, improved self‐management, and better engagement with care.

## Graphical abstract

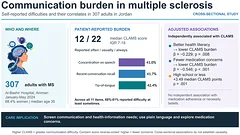

## 1 Introduction

Multiple sclerosis (MS) is a chronic immune‐mediated neurodegenerative disease affecting the central nervous system (CNS) and is considered one of the most prevalent chronic inflammatory diseases of the CNS, with an estimated global prevalence of more than 2 million cases (Feigin et al. 2017). In Jordan, the overall prevalence of MS is estimated at 24 per 100,000 based on national clinical data, placing the country within the low‐to‐moderate prevalence range compared with other countries in the Middle East and North Africa (MENA) region (Al‐Haddadin et al. 2021; Costa et al. 2025). The disease is characterized by progressive neurological disability resulting from inflammation, demyelination, and nerve degeneration (Filippi et al. 2018; A. J. Thompson et al. 2018). These disabilities are not limited to motor and sensory functions but also extend to cognitive impairment, with patients often experiencing slower information processing, dysarthria, and word‐finding difficulties (Yorkston et al. 2014). Collectively, these communication difficulties can negatively affect quality of life (QoL) by hindering the development of interpersonal relationships (Al‐Qerem et al. 2024; Al‐Qerem et al. 2026), restricting social participation, and limiting patient–physician communication. The availability of reliable tools to assess communication functioning in MS is therefore particularly important, given the continued under‐recognition of communication challenges in this population despite their well‐documented clinical and psychosocial impact (Baylor et al. 2011).

Focusing on the Jordanian context is important for both clinical and methodological reasons. MS care in Jordan is delivered within a healthcare system and sociocultural setting that shape access to disease information, patient–provider interaction, and treatment decision‐making, while Arabic‐language data on communication functioning in MS remain limited (Al‐Haddadin et al. 2021; Costa et al. 2025; Al‐Sharman et al. 2018). Examining these issues in Jordan therefore adds context‐specific evidence that may not be captured by data from Western cohorts alone.

The Communication and Language Assessment questionnaire for people with MS (CLAMS) (Al‐Qerem et al. 2025a) is a structured tool used to evaluate communication functioning in individuals with MS across several key domains, including expressive and receptive language, fluency, conversational competence, and communicative confidence (El‐Wahsh et al. 2020).

Effective MS management requires a comprehensive, multidimensional approach. Beyond neurological status, several behavioral factors that influence treatment outcomes warrant careful consideration. Medication adherence, in particular, is a key determinant of disease control, as disease‐modifying therapies play a central role in reducing relapse rates and improving long‐term prognosis (Giovannoni et al. 2016). Poor adherence has been associated with worse functional outcomes, increased hospitalizations, and higher relapse activity in MS (Menzin et al. 2015; Greene et al. 2022). The Medication Adherence Report Scale (MARS‐5) is a brief, validated self‐report instrument that is widely used to assess medication adherence across a range of chronic conditions, including MS (Horne and Weinman 1999).

Another important factor influencing MS management is health literacy, commonly defined as the ability to access, understand, evaluate, and use health information. Health literacy plays a central role in shaping health‐related behaviors, including self‐management, engagement in disease‐related decision‐making, and medication adherence (Sørensen et al. 2012). The Health Literacy Scale (HLS‐Q12) is a reliable and validated instrument used to assess health literacy across three key domains: health care, disease prevention, and health promotion (Jordan et al. 2011). Lower levels of health literacy have been consistently associated with suboptimal disease control and reduced involvement in treatment decisions (Dodson et al. 2015).

In addition to health literacy and adherence, patients’ beliefs about medications play a critical role in shaping treatment behavior. Patients often balance their perceived need for a medication (necessity beliefs) against concerns about potential side effects and dependency, a process described by the Necessity–Concerns Framework (Horne and Weinman 1999; Horne et al. 2013). The Beliefs about Medicines Questionnaire (BMQ) is a widely used instrument for assessing these beliefs (Horne and Weinman 1999). Previous studies have consistently shown that higher medication adherence is associated with stronger necessity beliefs and fewer concerns (Chan, Cooper, et al. 2020; Clatworthy et al. 2007).

Conceptually, the combined use of CLAMS, HLS‐Q12, BMQ, and MARS‐5 is grounded in chronic disease self‐management theory, which views self‐management as involving medical management, decision‐making, problem solving, resource use, and patient–provider partnership (Lorig and Holman 2003). It is also consistent with integrated health literacy models, which emphasize patients’ ability to access, understand, appraise, and apply health information (Sørensen et al. 2012), and with the Necessity–Concerns Framework, which links medication‐taking behavior to patients’ perceived need for treatment and concerns about treatment (Horne and Weinman 1999). Within this framework, communication difficulties may limit patients’ ability to ask questions, clarify instructions, and express treatment concerns, while limited health literacy and unresolved medication beliefs may reduce participation in decision‐making and medication‐related self‐management. Examining these measures together therefore allows communication functioning to be interpreted as part of the broader self‐management process rather than as an isolated symptom burden

Despite evidence that communication difficulties, health literacy, medication beliefs, and adherence each influence MS care (Giovannoni et al. 2016; Menzin et al. 2015; Greene et al. 2022) combined association remains insufficiently examined in Arabic‐speaking MS populations. This leaves a clear gap in understanding whether self‐perceived communication difficulties are independently associated with patients’ capacity to use health information, medication‐related beliefs, and adherence within a non‐Western clinical context, where MS care, access to disease information, and patient–provider communication may be shaped by local health‐system and sociocultural factors (Al‐Haddadin et al. 2021; Costa et al. 2025; Al‐Sharman et al. 2018). The present study therefore aimed to assess communication functioning using the CLAMS questionnaire and to examine its associations with MARS‐5, HLS‐Q12, and medication necessity and concern scores in patients with MS in Jordan.

## 2 Materials and Methods

### 2.1 Study Design and Participants

This cross‐sectional study included 307 patients with MS recruited from Al‐Bashir Hospital in Amman, Jordan. Data were collected between January and May 2024. Eligible participants were adults aged 18 years or older with a confirmed diagnosis of MS for at least 1 year. Patients’ medical records were reviewed by a trained researcher to obtain contact information, after which eligible individuals were contacted and invited to participate in the study. The study objectives were explained, and participants were informed that participation was voluntary and that they could withdraw at any time without consequence.

Informed consent was obtained electronically prior to questionnaire completion. All questionnaire items were set as mandatory fields, and therefore no missing data were observed. Ethical approval was obtained from the Ministry of Health (Reference No. MOH/REC/2021/263). The study was conducted in accordance with the ethical principles of the Declaration of Helsinki. Because recruitment was restricted to a single tertiary hospital and participation required voluntary completion, the sample may overrepresent patients who were more engaged with care.

### 2.2 Study Instruments

To assess participants’ ability to process health‐related information, the Arabic version of the Health Literacy Survey‐12 (HLS‐Q12) was used. This instrument is a shortened version of the European Health Literacy Survey Questionnaire and consists of 12 items rated on a four‐point Likert scale, ranging from 1 (“*very easy*”) to 4 (“*very difficult*”), with higher scores indicating greater difficulty and lower health literacy. The HLS‐Q12 assesses health literacy across three domains: health care, disease prevention, and health promotion. The Arabic version of the scale has undergone rigorous psychometric testing and has demonstrated good validity and reliability in the Jordanian context (Awwad et al. 2023).

The second tool used in the study was the BMQ (Horne and Weinman 1999), which assesses patients’ beliefs about their prescribed medications. The questionnaire comprises 10 items divided equally into two subscales, with all items rated on a five‐point Likert scale ranging from 1 (“*strongly disagree*”) to 5 (“*strongly agree*”). The Specific‐Necessity subscale (five items) evaluates the extent to which patients believe their medication is necessary for controlling their illness, with higher scores indicating stronger necessity beliefs. In contrast, the Specific‐Concerns subscale (five items) captures patients’ concerns about potential adverse effects associated with treatment. The Necessity–Concerns Differential, calculated as the difference between necessity and concern scores, has been shown to be a robust predictor of medication adherence. The Arabic version of the BMQ has been previously validated and has demonstrated good reliability and construct validity in the Jordanian population (Al‐Qerem et al. 2022).

The Medication Adherence Rating Scale (MARS‐5) was used to assess self‐reported adherence to prescribed medication, capturing both intentional and unintentional nonadherence (Chan, Horne, et al. 2020). The scale comprises five items evaluating medication‐taking behavior on a five‐point Likert scale, ranging from 1 (“*always*”) to 5 (“*never*”). All items are negatively worded; therefore, scores were reverse‐coded so that higher scores indicate better adherence. The validated Arabic version of the MARS‐5 was used in this study and is appropriate for assessing medication‐taking behavior in the study population (Al‐Qerem et al. 2022).

Finally, the Communication and Language Assessment Questionnaire for Multiple Sclerosis (CLAMS) was used to assess self‐reported communication difficulties among participants. This instrument is an 11‐item patient‐reported outcome measure that specifically evaluates cognitive–linguistic impairments in individuals with MS (El‐Wahsh et al. 2020). The locally validated Arabic version was used; this adaptation has demonstrated strong psychometric properties and cultural relevance for the study population (Al‐Qerem et al. 2025a). Items are rated on a four‐point Likert scale, with higher scores indicating greater severity of perceived communication and language difficulties. These instruments were analyzed together because they capture complementary domains of MS self‐management: communication functioning, capacity to obtain and use health information, cognitive beliefs about treatment, and medication‐taking behavior.

### 2.3 Sample Size

The final analysis included data from 307 participants, providing adequate statistical power for the planned analyses. This sample size exceeds the commonly cited psychometric guideline of a minimum of 10 participants per item for scale‐based analyses, corresponding to 110 participants for the 11‐item questionnaire (Mooi et al. 2010). The larger sample reduces the risk of sampling error and supports the stability and reliability of estimates related to communication and language difficulties.

### 2.4 Statistical Analysis

Statistical analyses were conducted using version 26 of the Statistical Package for the Social Sciences (SPSS). Categorical variables were summarized using frequencies and proportions, while continuous variables were described using the median and interquartile range (IQR). Internal consistency of the study scales was assessed using Cronbach's *α* coefficients.

Quantile regression was used to examine associations between independent variables and CLAMS scores. The comprehensive regression model incorporated HLS‐Q12 scores, MARS‐5 scores, age, duration of illness, sex, educational attainment, income level, marital status, employment status, Specific‐Necessity scores, and Specific‐Concern scores. Quantile regression was selected a priori because CLAMS scores were bounded and non‐normally distributed, this approach does not require normally distributed residuals and is less sensitive to skewed outcomes and extreme observations, making it appropriate for modeling the conditional median CLAMS score. Potential confounders were entered simultaneously based on clinical plausibility and prior literature in order to estimate the independent association of each variable with the median CLAMS score.

## 3 Results

The study included 307 individuals with MS, of whom 210 were female. The sociodemographic characteristics of the sample are presented in Table 1. Most participants were married (60.9%) and had a median age of 35 years (IQR: 28–44). Despite relatively high educational attainment, with 45.0% holding a bachelor's degree or higher and 22.1% having a diploma, the majority of participants (68.4%) were unemployed. Nearly three‐quarters of the sample (73.9%) reported a monthly income below 500 Jordanian dinars.

**TABLE 1:** Sociodemographic characteristics of the sample (*N* = 307).

Variable		*n* (%) or median (IQR)
Marital status	Married	187 (60.9%)
	Unmarried	120 (39.1%)
Education level	Bachelor's degree or higher	138 (45.0%)
	Diploma	68 (22.1%)
	High school or less	101 (32.9%)
Employment status	Employed	97 (31.6%)
	Unemployed	210 (68.4%)
Sex	Female	210 (68.4%)
	Male	97 (31.6%)
Income status (Jordanian dinars, JOD)	500 ≤	80 (26.1%)
	< 500	227 (73.9%)
Age		35 (28–44)

Table 2 summarizes participants’ responses to the MARS‐5. Overall, high levels of self‐reported adherence were observed across all items. Most participants reported that they never forgot to take their medications (60.3%), changed the prescribed dose (78.2%), stopped taking medication for a period of time (73.6%), intentionally skipped doses (77.5%), or took medication less than instructed (77.5%). Nonetheless, a minority of participants reported occasional or frequent nonadherence behaviors, most commonly forgetting doses and temporarily discontinuing medication. The median MARS‐5 score was 25 (IQR: 21–25) out of a maximum possible score of 25. The scale demonstrated high internal consistency, with a Cronbach's *α* of 0.86. Table A1 in the Supporting information lists the medications provided to patients with MS at Al‐Bashir Hospital.

**TABLE 2:** Patients’ responses to MARS‐5 items (frequency [%]).

	Always	Often	Sometimes	Rarely	Never
I forget to take them	9 (2.9%)	7 (2.3%)	44 (14.3%)	62 (20.2%)	185 (60.3%)
I change the dose	3 (1%)	9 (2.9%)	26 (8.5%)	29 (9.4%)	240 (78.2%)
I stop taking them for a while	10 (3.3%)	7 (2.3%)	32 (10.4%)	32 (10.4%)	226 (73.6%)
I decide to skip a dose	4 (1.3%)	10 (3.3%)	27 (8.8%)	28 (9.1%)	238 (77.5%)
I take medications less than instructed	8 (2.6%)	8 (2.6%)	24 (7.8%)	29 (9.4%)	238 (77.5%)

Patients’ health literacy levels, as measured by the HLS‐Q12, are presented in Table 3. Across most items, a substantial proportion of participants reported difficulty or great difficulty in accessing, understanding, and evaluating health‐related information. Tasks related to understanding medical emergencies, judging treatment options, following medication instructions, and interpreting preventive health information were particularly challenging. For example, more than three‐quarters of participants reported difficulty or great difficulty in understanding why health screenings are needed, and a similarly high proportion reported difficulty in making informed decisions to improve their health and well‐being. The median HLS‐Q12 score was 35 (IQR: 30–48) out of a maximum possible score of 48. The scale demonstrated acceptable internal consistency, with a Cronbach's *α* of 0.70.

**TABLE 3:** Patients' responses to HLS‐Q12 items (frequency [%]).

Items	Very easy	Easy	Difficult	Very difficult
Find information on treatments of illnesses that concern you?	83 (27%)	56 (18.2%)	51 (16.6%)	117 (38.1%)
To understand information about what to do in a medical emergency?	35 (11.4%)	72 (23.5%)	117 (38.1%)	83 (27%)
To judge the advantages and disadvantages of different treatment options?	38 (12.4%)	68 (22.1%)	118 (38.4%)	83 (27%)
Follow the instructions on medication?	49 (16%)	19 (6.2%)	129 (42%)	110 (35.8%)
Find information on how to manage mental health problems like stress and depression?	69 (22.5%)	75 (24.4%)	105 (34.2%)	58 (18.9%)
Understand why you need health screenings (e.g., breast exam, blood sugar test, blood pressure)?	23 (7.5%)	30 (9.8%)	119 (38.8%)	135 (44%)
Judge if the information in the media on health risks is reliable (TV, internet or other media)?	95 (30.9%)	63 (20.5%)	117 (38.1%)	32 (10.4%)
Decide how you can protect yourself from illness based on advice from family and friends?	37 (12.1%)	46 (15%)	131 (42.7%)	93 (30.3%)
Find information on health attitudes such as exercise, healthy food and nutrition?	33 (10.7%)	36 (11.7%)	143 (46.6%)	95 (30.9%)
Understand information on food packaging?	61 (19.9%)	53 (17.3%)	139 (45.3%)	54 (17.6%)
Judge which everyday behavior is related to your health (drinking and eating habits, exercise etc.)?	20 (6.5%)	51 (16.6%)	133 (43.3%)	103 (33.6%)
To make decisions to improve your health and well‐being?	26 (8.5%)	60 (19.5%)	114 (37.1%)	107 (34.9%)

Table 4 presents participants’ beliefs about medicines as assessed by the BMQ. Responses to the necessity items indicated relatively strong beliefs in the importance of medications, with most participants agreeing or strongly agreeing that their current and future health depended on their treatment and that medications protected them from disease worsening. At the same time, concerns about medication use were common. A substantial proportion of participants reported worries about long‐term effects, dependency, side effects, and disruption to daily life caused by medications. Cronbach's *α* values for the Necessity and Concern subscales were 0.85 and 0.65, respectively, indicating good internal consistency for the Necessity subscale and moderate consistency for the Concern subscale.

**TABLE 4:** Patients’ responses to BMQ items (frequency [%]).

	Strongly disagree	Disagree	Neutral	Agree	Strongly agree
Necessity scale					
My health, at present, depends on my medicine	22 (7.2%)	14 (4.6%)	54 (17.6%)	82 (26.7%)	135 (44%)
My medicine protects me from becoming worse	14 (4.6%)	9 (2.9%)	39 (12.7%)	114 (37.1%)	131 (42.7%)
My health in future will depend on my medicine	26 (8.5%)	14 (4.6%)	137 (44.6%)	87 (28.3%)	43 (14%)
My life would be impossible without my medicine	53 (17.3%)	42 (13.7%)	75 (24.4%)	64 (20.8%)	73 (23.8%)
Without my medicine, I would be very sick	28 (9.1%)	29 (9.4%)	71 (23.1%)	68 (22.1%)	111 (36.2%)
Concerns scale					
Having to take medicine worries me	34 (11.1%)	63 (20.5%)	63 (20.5%)	65 (21.2%)	82 (26.7%)
I sometimes worry about the long‐term effects of my medicine	23 (7.5%)	30 (9.8%)	36 (11.7%)	100 (32.6%)	118 (38.4%)
My medicines disrupt my life	28 (9.1%)	62 (20.2%)	131 (42.7%)	49 (16%)	37 (12.1%)
My medicines disrupt my life	103 (33.6%)	116 (37.8%)	51 (16.6%)	23 (7.5%)	14 (4.6%)
I sometimes worry about becoming too dependent on my medicine	26 (8.5%)	39 (12.7%)	54 (17.6%)	80 (26.1%)	108 (35.2%)
This medicine gives me unfavorable side effects	73 (23.8%)	75 (24.4%)	49 (16%)	61 (19.9%)	49 (16%)

Self‐reported communication and language difficulties, as assessed by the CLAMS, are presented in Table 5. The median CLAMS score was 12 (IQR: 7–18) out of a maximum possible score of 22. A large proportion of participants reported experiencing communication difficulties at least sometimes across all items. Difficulties related to word finding, maintaining a train of thought during speech, remembering information, and following conversations were particularly common, with approximately one‐third to more than 40% of participants reporting these problems often or always. Difficulties with organizing ideas logically, recalling recent conversations, and maintaining concentration during communication were also frequently reported, suggesting widespread perceived cognitive and linguistic difficulties within the study sample. The scale demonstrated acceptable reliability, with a Cronbach's *α* of 0.669.

**TABLE 5:** Reordered frequencies (%) of the Communication and Language Assessment questionnaire for Multiple Sclerosis (CLAMS).

Item	Never or rarely	Sometimes	Often/usually or always
Have difficulty thinking of the particular word you want?	69 (22.5%)	127 (41.4%)	111 (36.1%)
Have difficulty remembering your train of thought as you are speaking?	68 (22.1%)	119 (38.8%)	120 (39.1%)
Use a lot of vague or empty words such as “you know what I mean” instead of the right word?	98 (31.9%)	146 (47.6%)	63 (20.5%)
Find it difficult to remember information on the tip of your tongue?	59 (19.2%)	118 (38.4%)	130 (42.4%)
Find it difficult to convey precisely what you mean?	69 (22.5%)	120 (39.1%)	118 (38.4%)
Leave out important details?	83 (27%)	126 (41%)	98 (31.9%)
Find it difficult to concentrate enough to understand what is being said?	58 (18.9%)	123 (40.1%)	126 (41%)
Find it difficult to remember recent conversations?	69 (22.5%)	110 (35.8%)	128 (41.7%)
Find it difficult to keep track of the main details of conversations?	88 (28.7%)	118 (38.4%)	101 (32.9%)
Find it difficult to put ideas together in a logical way?	80 (26.1%)	107 (34.9%)	120 (39.1%)
Hesitate, pause, or repeat yourself?	80 (26.1%)	122 (39.7%)	105(34.2%)

Table 6 presents the quantile regression analysis examining factors associated with CLAMS scores. Lower health literacy scores were significantly associated with higher CLAMS scores, indicating greater communication difficulties (*β* = −0.229, *p* = 0.008). Higher medication concern scores were also significantly associated with increased communication difficulties (*β* = −0.546, *p* < 0.001). In practical terms, a 10‐point difference in HLS‐Q12 score corresponded to an estimated 2.29‐point difference in the median CLAMS score, while a 5‐point difference in concern score corresponded to an estimated 2.73‐point difference. These shifts suggest small‐to‐moderate changes in self‐reported communication difficulty, although formal clinical cut‐points for CLAMS are not established. Marital status showed a significant association, with unmarried participants reporting lower CLAMS scores than married participants (*β* = −2.298, *p* = 0.018). Educational level was a further significant predictor, with participants who had a high school education or less showing significantly higher CLAMS scores than those with a university or college education (*β* = 3.486, *p* = 0.001). Other variables, including age, duration of illness, medication adherence, necessity beliefs, sex, employment status, and income level, were not significantly associated with communication functioning in the adjusted model.

**TABLE 6:** Quantile regression results showing variables significantly associated with CLAMS.

				95% confidence interval	
		*Β*	*p* value	Lower bound	Upper bound
(Intercept)		21.142	0.000	12.678	29.606
Age		−0.044	0.306	−0.128	0.040
Duration of illness		0.004	0.119	−0.001	0.010
MARS‐5 score		0.190	0.140	−0.062	0.443
HLS‐Q12 score		−0.229	0.008	−0.398	−0.060
Concerns score		−0.546	0.000	−0.749	−0.342
Necessity score		0.141	0.152	−0.052	0.335
Gender	Female	0.197	0.842	−1.745	2.139
	Male	Reference group			
Marital status	Other	−2.298	0.018	−4.199	−0.396
	Married	Reference group			
Employment status	Unemployed	−0.431	0.678	−2.475	1.613
	Employed	Reference group			
Income status	< 500 JDs*	1.906	0.081	− 0.235	4.048
	⇒500 JDs	Reference group			
Education Level	High school or less	3.486	0.001	1.361	5.610
	University/college	Reference group			

Abbreviation: *β*, Coefficient.

* JDs = Jordanian Dinars, 1 Jordanian Dinar equals 1.41 United States Dollar.

## 4 Discussion

This study examined communication functioning among Jordanian adults with MS and its associations with sociodemographic characteristics, medication adherence, health literacy, and medication beliefs. Communication difficulties were common, and lower health literacy and greater medication concerns were independently associated with poorer communication functioning. These findings suggest that communication in MS is shaped by cognitive and psychosocial factors relevant to self‐management. Because the design was cross‐sectional, the findings should be interpreted as associations rather than causal effects.

The high prevalence of communication difficulties observed in this study is consistent with previous research showing that individuals with MS commonly experience challenges related to expressive and receptive language, memory retrieval, and conversational processing (Plotas et al. 2023; Lebkuecher et al. 2021). Because CLAMS is a patient‐reported measure, the high frequency of communication difficulties observed in this study should be interpreted as perceived communication burden rather than as evidence of objectively measured speech, language, cognitive, or neuropsychological impairment. These difficulties can restrict communicative participation, making it harder for patients to maintain relationships, fulfill work roles, and manage everyday interactions, particularly when fatigue and cognitive impairment are present (Yorkston et al. 2014). Beyond interpersonal communication, people with MS have also been shown to experience difficulties when interacting with health care providers, where complex medical information and shared decision‐making are central to effective care and disease management (C. M. Thompson et al. 2022). An observational study conducted in Australia further highlighted these challenges, with participants identifying unmet needs related to managing communication changes. Many emphasized the importance of clinicians initiating conversations about communication difficulties and supporting collaborative care through patient education and timely referral to appropriate services (El‐Wahsh et al. 2022). Communication impairments, although often under‐recognized, may therefore limit patients’ ability to navigate health care systems, understand and act on treatment instructions, and communicate concerns or needs effectively, all of which are critical for optimal self‐management.

Health literacy emerged as an important determinant of communication functioning in this study. This finding is consistent with existing evidence showing that individuals with chronic illnesses who have limited health literacy often experience greater difficulty understanding their condition, evaluating treatment options, and engaging effectively with health care professionals (Gazmararian et al. 2003; Wynia and Osborn 2010). The challenges identified in our sample, including difficulties interpreting medical guidance and judging health information, have also been reported in other MS populations. For example, a recent study of e‐health literacy among people with MS in Lebanon found that patients struggled to assess the credibility of online health information and had difficulty navigating complex medical terminology, conflicting information, and non‐disease‐specific resources. These challenges were identified as barriers to effective communication and informed decision‐making (Lteif et al. 2025).

Similar patterns have been observed in other studies of people with MS, where lower health literacy was associated with reduced engagement in disease management and care processes (Marrie et al. 2014; Dehghani 2021). Conceptually, this association can be interpreted through Sørensen et al.’s integrated model of health literacy, which defines health literacy as the capacity to access, understand, appraise, and apply health information in health‐care contexts (Sørensen et al. 2012), and through Paasche–Orlow and Wolf's causal pathways model, which positions the patient–provider relationship and self‐care as key mechanisms linking health literacy to health outcomes (Paasche‐Orlow and Wolf 2007). Within this framework, communication functioning and health literacy can be viewed as interdependent rather than strictly sequential constructs: communication difficulties may reduce patients’ ability to ask clarifying questions, process spoken or written information, and retain treatment instructions, while limited health literacy may reduce confidence and effectiveness during clinical communication (Ishikawa and Kiuchi 2010; El‐Wahsh et al. 2022; McKenna and Gilheaney 2024). The relationship may therefore be bidirectional, and the cross‐sectional design does not allow the temporal ordering of communication difficulties and health literacy to be determined.

Medication beliefs were also significantly associated with communication difficulties in this study. Although many participants expressed strong beliefs in the necessity of their treatments, concerns about side effects, long‐term dependency, and the impact of medications on daily life were commonly reported. Similar findings have been documented in studies of MS and other chronic conditions, where negative perceptions of medications have been linked to poorer adherence, supporting the presence of these mixed or conflicting beliefs. For example, a study of individuals with relapsing remitting MS found that patients who viewed medications as overused or harmful were more likely to intentionally deviate from prescribed treatment, suggesting that negative medication beliefs can influence engagement with treatment regimens (Kołtuniuk et al. 2023). Similarly, a prospective study of people with MS reported that perceptions of medication harm and overuse were significant predictors of both adherence and persistence with disease‐modifying therapies over time (Neter et al. 2021). This pattern of strong necessity beliefs alongside elevated concerns indicates that interventions should address not only factual knowledge about treatment, but also the emotional and cognitive aspects of medication use, to support both adherence and communication confidence.

The high adherence observed despite only intermediate health literacy may reflect the treatment context of this sample, in which a substantial proportion of patients received monthly, semi‐annual, or otherwise supervised therapies (Table A1 in the Supporting Information). This interpretation is consistent with a related Jordanian MS analysis showing that better health literacy, stronger necessity beliefs, and less frequent dosing were associated with higher adherence (Al‐Qerem et al. 2025b). Adherence remains central to MS follow‐up and surveillance because consistent use of disease‐modifying therapies and reliable attendance to treatment schedules influence how clinicians interpret relapse activity, adverse effects, MRI monitoring, and disease progression. The present findings should therefore be interpreted in light of treatment delivery patterns and patient engagement with care, rather than as evidence that health literacy is unimportant for adherence. Similar concerns have been noted in screening and surveillance research, where adherence to recommended pathways can influence program efficiency and the interpretation of follow‐up outcomes (Y. J. Wu et al. 2024)

The associations between sociodemographic factors and communication functioning observed in our regression model are broadly consistent with previous research. Lower educational attainment has been linked to poorer cognitive performance and reduced verbal fluency in people with MS, both of which can negatively affect communication processes, including expressive and receptive language functioning (Estrada‐López et al. 2021). In addition, a systematic review examining socioeconomic and demographic influences on interpersonal communication in chronic illness found that lower education and health literacy were associated with less effective patient–provider communication. This supports the broader role of sociodemographic factors in shaping communication functioning in conditions such as MS (Svyntozelska et al. 2025). Although income was not independently associated with communication scores in the current model, socioeconomic disadvantage may still influence MS care indirectly through treatment access, continuity of care, and navigation of health services (Dobson et al. 2022; F. Z. Wu and Chang 2023).

Although relatively little is known about the relationship between marital status and communication functioning in MS, earlier studies have reported poorer social engagement and reduced interaction with health care services among unmarried individuals with MS (Hajric et al. 2022). This contrasts with the findings of the present study, in which unmarried participants reported lower CLAMS scores, indicating fewer perceived communication difficulties. This difference may reflect variation in outcome measures, as CLAMS assesses perceived communication difficulties rather than broader indicators of social functioning or QoL. Together, these findings highlight the complexity of sociodemographic influences on communication functioning and suggest that marital status may shape different aspects of the lived experience in distinct ways.

Our findings are consistent with broader literature on psychosocial adjustment and QoL in MS. A study involving 160 individuals with MS reported that difficulties in psychosocial adjustment were strongly associated with poorer health‐related QoL and accounted for substantial variation in both mental and physical health outcomes after controlling for demographic factors (Hyarat et al. 2019). This aligns with the present findings by highlighting the importance of cognitive and psychosocial dimensions, including health literacy, communication functioning, and beliefs about medications, in shaping overall health outcomes in MS. Difficulties in communication and limited health literacy may hinder psychosocial adjustment, contributing to greater distress, reduced QoL, and challenges in managing health and treatment.

Overall, this study highlights the multidimensional nature of communication difficulties in MS and suggests that addressing health literacy and medication beliefs may improve patients’ ability to engage in effective communication, with potential benefits for self‐management and QoL. From a practical perspective, these findings support a staged implementation pathway in MS clinics. Brief screening with CLAMS and HLS‐Q12 could be incorporated into routine follow‐up visits to identify patients who struggle with communication or with understanding health information (Al‐Qerem et al. 2025a). Patients with high CLAMS scores could be considered for speech‐language assessment and, where cognitive concerns are evident, neuropsychological evaluation (Warde et al. 2018; Meca‐Lallana et al. 2021; El‐Wahsh et al. 2022). Patients with lower health literacy may benefit from plain‐language counselling and teach‐back strategies (Ishikawa and Kiuchi 2010; El‐Wahsh et al. 2022; McKenna and Gilheaney 2024). Structured pharmacist‐ or nurse‐led medication discussions may help elicit modifiable barriers to adherence, including necessity beliefs and medication concerns (Ben‐Zacharia et al. 2018; Neter et al. 2021) More broadly, as highlighted in other areas of screening and risk management, interventions are often more effective when they are tailored to the characteristics and context of the target population (Y. J. Wu and Chang 2023).

Future research should examine targeted educational and psychosocial interventions designed to support people with MS in navigating complex health information and treatment decisions.

### 4.1 Strengths, Limitations, and Future Directions

To our knowledge, this is the first study to comprehensively examine communication functioning among adults with MS in Jordan by exploring its associations with health literacy, medication adherence, beliefs about medications, and sociodemographic factors. By considering these factors together, the study offers a multidimensional perspective that reflects the complexity of living with MS.

Several limitations should nevertheless be acknowledged. The cross‐sectional design limits the ability to draw causal inferences about the relationships between communication functioning, health literacy, medication beliefs, and adherence. Recruitment from a single tertiary center and voluntary survey participation may limit representativeness, and the findings should not be assumed to be directly generalizable to all people with MS in Jordan or to health systems with different models of MS care. In addition, reliance on patient‐reported instruments may have introduced recall error, social desirability bias, and variation in participants’ willingness to disclose communication difficulties or medication‐taking behaviors. Accordingly, the high prevalence of communication difficulties should be interpreted as perceived communication burden rather than clinician‐rated or objectively tested impairment. The Cronbach's *α* values for CLAMS and the BMQ Concerns subscale were slightly below the conventional 0.70 threshold, which may have modestly reduced measurement precision and should be considered when interpreting associations involving these measures. Finally, key clinical and neuropsychological variables, such as MS subtype, disease severity, and objective cognitive assessments, were not included, although these may have provided further insight into the mechanisms underlying communication difficulties. Future research should use longitudinal designs to explore changes in communication functioning over time and their relationship with cognitive decline, disease progression, and treatment experiences. The inclusion of objective measures of cognitive and language functioning alongside self‐reported outcomes may also strengthen future studies and improve understanding of communication difficulties in MS.

## 5 Conclusion

This study provides important insight into communication functioning among Jordanian adults living with MS and highlights its associations with health literacy, medication beliefs, and selected sociodemographic factors. The findings indicate that communication difficulties are common and have a meaningful impact on patients’ ability to understand health information, evaluate treatment‐related risks and benefits, and engage effectively with health care providers. Lower health literacy and greater concerns about medications emerged as key contributors to poorer communication functioning, underscoring the importance of cognitive and psychosocial factors in shaping everyday communication experiences in MS. Overall, these results suggest that improving communication outcomes in MS requires a holistic approach that goes beyond symptom management and includes strategies to strengthen health literacy, address medication‐related concerns, and support meaningful patient–provider interactions.

## Author Contributions

Conceptualization: **Walid Al‐Qerem** and **Sawsan Khdair**. Methodology: Walid Al‐Qerem. Software: **Judith Eberhardt**. Validation: Walid Al‐Qerem and Sawsan Khdair. Formal analysis: Walid Al‐Qerem. Investigation: **Dunia Basem**. Data curation: Walid Al‐Qerem and **Lama Sawaftah**. Supervision: Judith Eberhardt. Project administration: Walid Al‐Qerem. Writing – original draft and review and editing: All authors. All authors have read and agreed to the published version of the manuscript.

## Funding

The authors have nothing to report.

## Ethics Statement

Ethical approval was obtained from the Ministry of Health (Reference No. MOH/REC/2021/263). All participants provided informed consent.

## Conflicts of Interest

The authors declare no conflicts of interest.

## Supporting information




**Supplementary Material**: brb371749‐sup‐0001‐SuppMat.docx

**Supplementary Table**: brb371749‐sup‐0002‐SuppMat.docx

## Data Availability

The data supporting the findings of this study are available from the corresponding author upon reasonable request.
