# Supplementary material for: Communication Difficulties and Associations With Health Literacy, Medication Beliefs, and Adherence in Multiple Sclerosis
Source: Brain Behav. 2026 Sep 7;16(9):e71749. doi: 10.1002/brb3.71749 (PMC13550869; doi:10.1002/brb3.71749)
Supplement: Supplementary file 1 [file BRB3-16-e71749-s001.docx]

STROBE Statement—Checklist of items that should be included in reports of ***cross-sectional studies***

|  | Item No | Recommendation | Page No |
| --- | --- | --- | --- |
| **Title and abstract** | 1 | (*a*) Cross-sectional study | 2 |
|  |  | (*b*) abstract | 2 |
| Introduction | | | |
| Background/rationale | 2 | Introduction | 2-4 |
| Objectives | 3 | Introduction | 4 |
| Methods | | | |
| Study design | 4 | Methods – Study design and participants | 4 |
| Setting | 5 | Methods – Study design and participants | 4 |
| Participants | 6 | (*a*) Methods – Study design and participants | 4 |
| Variables | 7 | Methods – Study instruments | 4-5 |
| Data sources/ measurement | 8* | Methods – Study instruments | *4-5* |
| Bias | 9 | Discussion – Limitations | 16 |
| Study size | 10 | Methods – Sample size | 5 |
| Quantitative variables | 11 | Methods – Statistical analysis | 6 |
| Statistical methods | 12 | (*a*) Methods – Statistical analysis | 6 |
|  |  | (*b*) Methods – Statistical analysis | 6 |
|  |  | (*c*) Methods – Study design and participants | 4 |
|  |  | (*d*) Methods – Statistical analysis | 6 |
|  |  | (*e*) Methods – Statistical analysis | 6 |
| Results | | | |
| Participants | 13* | (a) results | 6 |
|  |  | (b) N/A |  |
|  |  | (c) N/A |  |
| Descriptive data | 14* | (a) results | 6 |
|  |  | (b) N/A |  |
| Outcome data | 15* | Tables | 17-21 |
| Main results | 16 | (*a*) Table 6 | 21 |
|  |  | (*b*) Tables 1 & 6 | 17&21 |
|  |  | (*c*) N/A |  |
| Other analyses | 17 | Regression model | 7 |
| Discussion | | | |
| Key results | 18 | Discussion | 8 |
| Limitations | 19 | Limitations | 9 |
| Interpretation | 20 | discussion | 8-9 |
| Generalisability | 21 | N/A |  |
| Other information | | | |
| Funding | 22 | Funding | 1 |

*Give information separately for exposed and unexposed groups.

**Note:** An Explanation and Elaboration article discusses each checklist item and gives methodological background and published examples of transparent reporting. The STROBE checklist is best used in conjunction with this article (freely available on the Web sites of PLoS Medicine at http://www.plosmedicine.org/, Annals of Internal Medicine at http://www.annals.org/, and Epidemiology at http://www.epidem.com/). Information on the STROBE Initiative is available at www.strobe-statement.org.
