# Supplementary material for: Communication Difficulties and Associations With Health Literacy, Medication Beliefs, and Adherence in Multiple Sclerosis
Source: Brain Behav. 2026 Sep 7;16(9):e71749. doi: 10.1002/brb3.71749 (PMC13550869; doi:10.1002/brb3.71749)
Supplement: Supplementary file 2 [file BRB3-16-e71749-s002.docx]

Table A1. Available medications for MS patients in Al-Basher Hospital

| Medication | Route of administration | Dosage frequency | Frequency (%) |
| --- | --- | --- | --- |
| Dimethyl fumarate tab | oral | Twice a day | 101 (32.9%) |
| Fingolimod 0.5 mg | oral | Once a day | 53 (17.3%) |
| Natalizumab 300 mg/15ml | infusion | Once a month | 16 (5.2%) |
| Ocrelizumab 300 mg /15ml | infusion | Twice a year | 31 (10.1%) |
| Ofatumumab 20 mg | subcutaneously | Once a month | 124 (40.4%) |
